# Dihydrotestosterone Induces Arterial Stiffening in Female Mice

**DOI:** 10.21203/rs.3.rs-2522089/v1

**Published:** 2023-02-07

**Authors:** Alec C. Horton, Mary M. Wilkinson, Isabella Kilanowski-Doroh, Benard O. Ogola, Sarah H. Lindsey

**Affiliations:** Tulane University Department of Pharmacology and the Tulane Brain Institute, New Orleans, LA; Tulane University Department of Pharmacology, New Orleans, LA; Tulane University Department of Pharmacology, New Orleans, LA; Vascular Biology Center and Department of Medicine, Medical College of Georgia at Augusta University, Augusta, GA; Tulane University Department of Pharmacology, the Tulane Brain Institute, and the Tulane Center of Excellence in Sex-Based Biology and Medicine, New Orleans, LA

**Keywords:** Testosterone, arterial stiffness, gender-affirming therapy, hormones, estrogen receptors, polycystic ovarian syndrome

## Abstract

**Background:**

Testosterone is the predominant sex hormone in men and is increased in women with polycystic ovarian syndrome. These patients also experience an increased risk for cardiovascular diseases including hypertension and arterial stiffness. Since our previous work shows an important role for the G protein-coupled estrogen receptor (GPER) in arterial stiffness, we hypothesized that other hormones including androgens may impact arterial stiffness in female mice via regulation of GPER.

**Methods:**

The impact of the non-aromatizable androgen dihydrotestosterone (DHT), the glucocorticoid dexamethasone, and the progestin medroxyprogesterone acetate (all 100 nM for 24 h) on GPER and ERα expression was assessed in cultured vascular smooth muscle cells using droplet digital PCR (ddPCR). To assess the *in vivo* impact of the DHT-induced downregulation of GPER, female ovary-intact C57Bl/6 mice were treated with silastic capsules containing DHT for 4 weeks, one with a dosage expected to mimic human male DHT levels and another to double the expected human concentration (n=8–9/group).

**Results:**

GPER mRNA was only decreased by DHT (P=0.001), while ERα expression was significantly suppressed by all hormones (P<0.0001). While blood pressure was not different between groups (P= 0.59), there was a dose-dependent increase in body weight (control 22±2 g, single dose 24±2 g, double dose 26±2 g; P=0.0002). Intracarotid stiffness measured via pulse wave velocity showed a more than two-fold increase in both DHT-treated groups (control 1.9±0.3 m/s, single dose 4.3±0.8 m/s, double dose 4.8±1.0 m/s). Histological analysis of aortic sections using Masson’s trichrome showed a significant decrease in collagen between the control group (24 ± 5%) and the double dose group (17 ± 3%, P=0.007), despite no changes in aortic wall thickness or smooth muscle content. Lastly, ddPCR showed that *in vivo* DHT treatment decreased aortic expression of both GPER (control 20±5, single dose 10.5 ± 5.6, double dose 10±4 copies/ng; P=0.001) and ERα (control 54±2, single dose 24±13, and double dose 23 ± 12 copies/ng; P=0.003).

**Conclusions:**

These findings indicate that testosterone promotes arterial stiffening and cardiovascular damage in female mice and is associated with decreased estrogen receptor expression. These data are important not only for polycystic ovarian syndrome patients but also women using testosterone for fitness, gender transitioning, or reduced libido.

## Background

Cardiovascular disease (CVD) is the leading cause of mortality worldwide and is sexually dimorphic in not only incidence but in presentation and diagnosis [1]. While many variables contribute to CVD including genetics, age, activity level, diet, substance use, and sleep, it is well known that sex hormones play a critical role in sex differences in CVD [2, 3]. While many studies focus on the role of estrogen in females and testosterone in males, these steroid hormones differ by only one enzymatic step and are present in the circulation of both sexes. In fact, throughout the female lifespan levels of circulating testosterone are higher than estradiol, although still lower than testosterone levels in men [4]. The role of sex hormones becomes even more important when considering that aging dramatically changes levels of both estrogen and testosterone [5, 6]. How these hormonal changes impact the progression of CVD is still not fully understood.

Sex hormone levels are also disrupted in certain endocrine disorders such as polycystic ovarian syndrome (PCOS), which affects 20% of women of reproductive age worldwide [7]. Because of the hyperandrogenism often associated with PCOS, women with the disorder can experience stereotypical symptoms such as increased acne and body hair, infertility, and weight gain [8]. Women suffering from PCOS are also at higher risk of developing a variety of comorbidities including hypertension, diabetes, arterial stiffness, and atherosclerosis [9]. While some lifestyle and pharmacological approaches can reduce risk, additional knowledge is needed on the molecular mechanisms that connect PCOS to these diseases.

Our previous work shows an important role for the novel G protein-coupled estrogen receptor (GPER) in both hypertension and arterial stiffening, both strong risk factors for cardiovascular mortality [10]. We previously showed that estrogen is necessary for female protection from angiotensin II-dependent hypertension in a transgenic rat model, while selective activation of GPER provides the same antihypertensive effect as nonselective estradiol treatment [11, 12]. Moreover, we find that genetic deletion of GPER in mice induces arterial stiffness [13–15]. Considering these findings, we hypothesized in the current study that testosterone may counteract the protective effects of GPER on arterial stiffness due to regulation of vascular receptor expression. We utilized dihydrotestosterone (DHT) to avoid aromatization to estrogen and direct binding to GPER as well as the nuclear estrogen receptors alpha (ERα) and beta (ERβ). We determined the impact of DHT treatment both *in vitro* and *in vivo* on the vascular expression of GPER and ERα, which we previously showed were the dominant receptor subtypes in aortic tissue [16, 17].

## Methods

### Cell Culture.

Mouse aortic smooth muscle cells (MOVAS; ATCC CRL-2797) or rat aortic smooth muscle cells (A7r5; ATCC CRL-1444) were grown in 10% FBS media to 80% confluency and then starved for 24 h in charcoal-stripped 0.5% FBS. The plates were treated for 24 h with 100 nm of dexamethasone (DEX), dihydrotestosterone (DHT), or medroxyprogesterone acetate (MPA) or vehicle. After treatment, RNA was extracted, and droplet digital PCR (ddPCR) was used to quantify the expression of both GPER and ERα. Results were analyzed using a one-way ANOVA.

### Animals and Drug Treatment.

All procedures were approved by the Tulane University Animal Care and Use Committee. Female C57BL/6 mice were received from Jackson Laboratories at 10 weeks of age. At 15–16 weeks of age, mice were randomized to one of three treatments administered via subcutaneous silastic capsule for 4 weeks: control (CON; empty silastic capsule), group I (1 capsule containing 5 mg of DHT), or group II (2 capsules containing 5 mg DHT). The 5 mg dose of DHT was selected based on previous studies showing it produces plasma concentrations of roughly 30 ng/dl which is in the range for adult men (14–77 ng/dl) but higher than average for adult women (2.6–26 ng/dl) [18, 19]. Body weight was recorded prior to capsule implantation and again just prior to sacrifice. The uterus and heart were also weighed post-sacrifice. Aortas were halved and preserved in either RNALater (top half) for RNA extraction and droplet digital PCR or formalin (bottom half) for aortic morphology and Masson’s trichrome (MTC) staining. Blood was collected by cardiac puncture and subjected to DHT ELISA (IBL America #IB59116) according to the product instructions.

### Cardiovascular measurements.

Blood pressure was obtained via tail cuff plethysmography using the Kent Scientific CODA Volume Pressure Recording system. Blood pressure was measured at baseline before the initiation of treatment and at weekly intervals during treatment. Each mouse received two training days before testing day. No anesthesia was used on any training or testing day. Within each session, 15 cycles were obtained with the first 5 considered acclimation. The last 10 readings were used to compute the mean as long as tail blood volume was a minimum of 20 μl and the pressure curve was the appropriate shape. Intracarotid pulse wave velocity (PWV) was measured as previously described in anesthetized mice using the Visual Sonics Vevo 3100 high resolution ultrasound [14].

### Droplet digital PCR (ddPCR).

Aortas were stored in RNAlater before being mechanically homogenized and extracted using the RNeasy Micro Kit (Qiagen #74004). Validated mouse primers for GPER and ERα (Biorad dMmuCPE5103030 and dMmuCPE5092741) along with the protocol for ddPCR were previously described [16, 17].

### Aortic Histology.

Aortas were fixed in formalin for 24 h before being transferred to 70% ethanol, formalin-embedded, and sectioned onto slides. Masson’s Trichrome staining (Newcomer Supply #1403) was carried out according to the manufacturer’s protocol. Image-J was used for analysis of geometry and area fraction of staining for smooth muscle and collagen.

### Pulse Wave Velocity.

Arterial stiffness was assessed via intracarotid pulse wave velocity using a VisualSonics Vevo 1100 high-frequency ultrasound system with MS400 (18–38 mHz) linear probe. As previously described, the carotid artery was imaged in B-mode from the aortic arch to the bifurcation and arrival of the pulse wave was measured in Doppler mode [14].

### Statistical Analysis

Cell culture experiments in MOVAS cells and all data from the *in vivo* study were analyzed by one-way ANOVA with Dunnett’s multiple comparisons test. Data from A7r5 cells was analyzed using two-way ANOVA with Sidak’s post-hoc tests.

## Results

**Figure 1.**
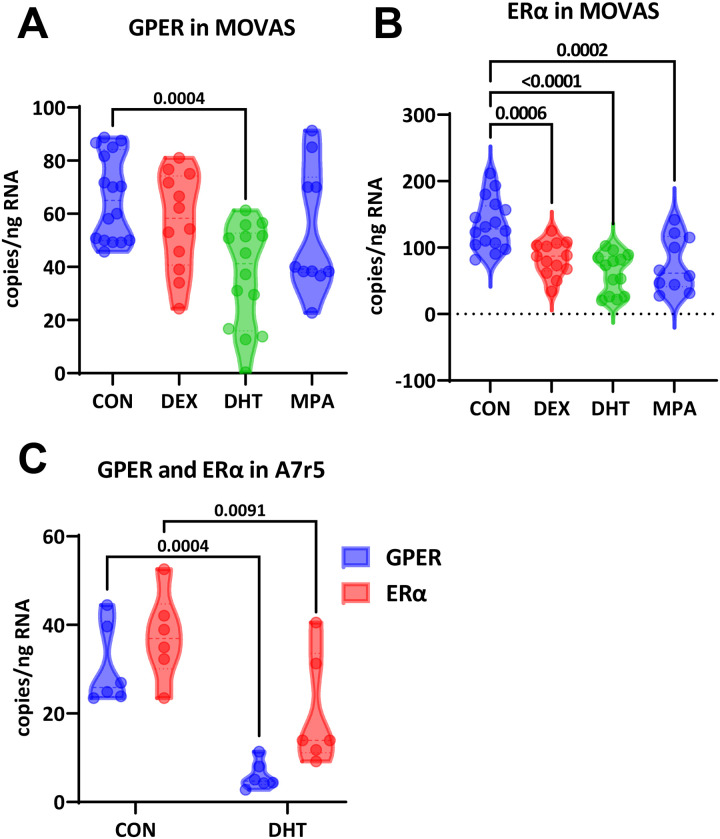
Impact of *in vitro* hormones on estrogen receptor expression in vascular smooth muscle cells. DMSO vehicle (CON), dexamethasone (DEX), dihtdrotestosterone (DHT0 and medroxyprogesterone acetate (MPA) was added to charcoal-stripped and pheno red-free media at a concentration of 100 nM for 24 h. **(A)** In mouse aortic smooth muscle cells (MOVAS), GPER expression was significantly decreased only by DHT (One-way ANOVA, P=0.002; Dunnett’s post-hoc test, N=10–16). **(B)** ERα was significantly downregulated in MOVAS by all three hormones (One-way ANOVA, P<0.0001; Dunnett’s post-hoc test, N=10–16). **(C)** In the rat aortic smooth muscle cell line (A7r5), DHT significantly supressed mRNA for both GPER and ERα (Two-way ANOVA, P<0.0001, Sidak’s post-hoc test, N=6).

Expression of GPER in mouse vascular smooth muscle cells (MOVAS) was significantly decreased by DHT (66±4 vs. 37±5 copies/ng RNA, P=0.0004, n=12–14) but neither DEX (57±5 copies/ng RNA, P=0.49, n=12) nor MPA (53±8 copies/ng RNA, P=0.25, n=10; Figure 1A). In contrast, ERα mRNA was decreased by DEX (135±9 vs. 84±7 copies/ng RNA, P=0.0006, n=13–16), DHT (62±8 copies/ng RNA, P<0.0001, n=13), and MPA (75±13 copies/ng RNA, P=0.0002, n=10; Figure 1B). The impact of DHT on GPER and ERα was also tested in the A7r5 rat aortic smooth muscle cell line. Again, DHT induced significant downregulation of both GPER (31±4 vs. 6±1 copies/ng RNA, P=0.0004, n=6) and ERα (37±4 vs. 20±5 copies/ng RNA, P=0.009, n=6; Figure 1C). Gene expression of ERβ was not tested because we previously showed it was below the level of detection in both rat and mouse aorta [16, 17].

**Figure 2.**
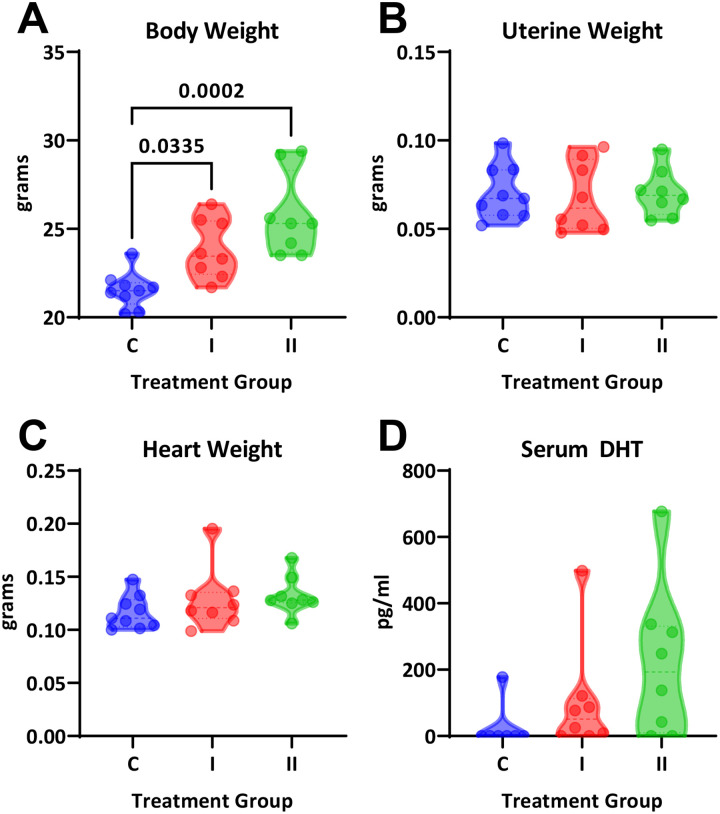
Physiological parameters in female mice treated with DHT. Intact female mice were randomized to three treatment groups: control (C), one capsule of DHT (I) or two capsules of DHT (II). **(A)** DHT increased body weight in a dose-dependent manner (One-way ANOVA, P=0.0002; Sidak’s post-hoc test, N=8–9). Neither **(B)** uterine weight or **(C)** heart weight were different across group (One-way ANOVA, P>0.05). **(D)** Serum concentrations of DHT showed statistical significance for linear trend (P=0.04).

**Figure 3.**
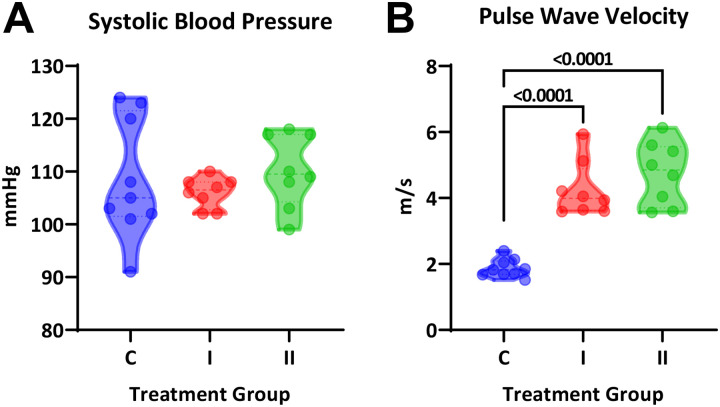
Cardiovascular parameters in female mice treated with DHT. **(A)** Systolic blood pressure was similar across groups (One-way ANOVA, P=0.59). **(B)** Pulse wave velocity was significantly increased in both DHT-treated groups (One-way ANOVA, P<0.0001, Dunnett’s post-hoc test).

*In vivo* treatment with DHT increased body weight in a dose-dependent manner but did not impact heart or uterine weight (Figure 2A–C). Serum DHT measured by ELISA showed wide variability, most likely due to inconsistencies in blood collection, but showed an upward trend with treatment groups (one-way ANOVA, P=0.11; test for linear trend, P=0.04; Figure 2D). Systolic blood pressure was not significantly different between groups and throughout the study (one-way ANOVA, P=0.59; Figure 3A). Intracarotid pulse wave velocity, a measure of arterial stiffness, was twice as high in both treatment groups compared with the control group (1.9±0.09, 4.3±0.3, and 4.8±0.3 m/s; P<0.0001; Figure 3B).

**Figure 4.**
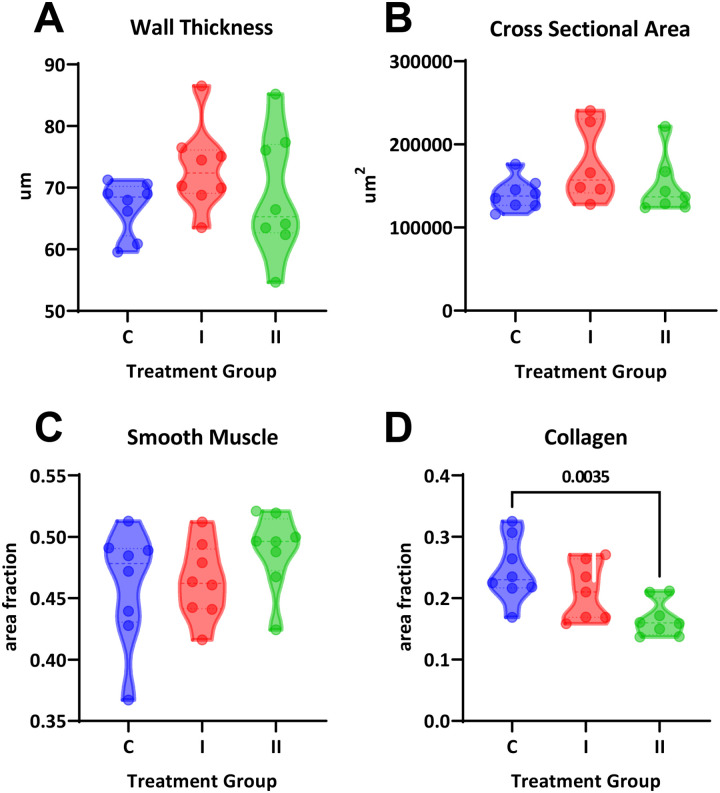
Histological analysis of aortic cross-sections. **(A)** Aortic wall thickness, **(B)** aortic cross-sectional area, and **(C)** smooth muscle content were not different across groups (One-way ANOVA, P>0.05). **(D)** Collagen content was significantly decreased in the high dose DHT group (One-way ANOVA, P=0.007, Dunnett’s post-hoc test).

To assess whether the arterial stiffness was associated with changes in vascular fibrosis, aortic sections were stained with Masson’s Trichrome. No differences were observed in aortic wall thickness (P=0.24; Figure 4A) or cross-sectional area (P=0.18; Figure 4B). Smooth muscle content was also not impacted by DHT treatment (P=0.26; Figure 4C). Surprisingly, we found significantly lower vascular collagen content at the high dose of DHT (P=0.003; Figure 4D).

**Figure 5.**
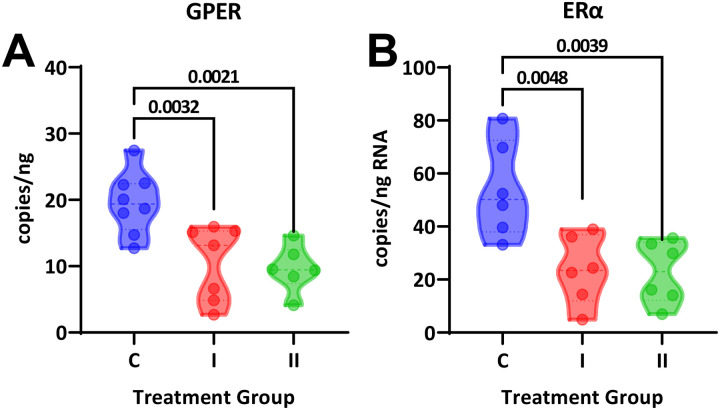
RNA analysis of estrogen receptors in aorta. Both **(A)** GPER and **(B)** ERα were significantly downregulated in aortic tissue from both DHT-treated groups (One-way ANOVA, P<0.01, Dunnett’s post-hoc test).

Lastly, vascular estrogen receptor mRNA was analyzed in aortic samples. Both DHT treatment groups showed lower copy numbers for both GPER and ERα in comparison with controls (Figure 5). GPER was decreased from 20±2 copies/ng RNA in controls to 11±2 copies (P=0.003) in the low dose group and 10±1 copies/ng RNA (P=0.002) in the high dose group. ERα levels were at 54±7 copies/ng RNA in the control group and decreased to 24±5 (P=0.005) and 23±5 (P=0.004) in the I and II capsule groups, respectively.

## Discussion

The current study showed that both *in vitro* and *in vivo* treatment with DHT downregulates vascular expression of GPER and ERα. Moreover, we found that this is associated with increased arterial stiffness in adult female mice. This study confirms that crosstalk or feedback loops exist between different sex hormones so that altered levels of one hormone may impact signaling of another and underscores the importance of assessing the impact of hormones together rather than separately. Taken with our previous work showing that GPER deletion increases vascular stiffness, these data underscore the importance of GPER signaling on vascular health. Additional work is needed to fully elucidate the specific cellular mechanisms involved in GPER’s protection from vascular stiffening.

In cultured vascular smooth muscle cells, DHT decreased the expression of GPER, while DEX, DHT, and MPA downregulated ERα. These results suggest that ERα levels are sensitive to androgens, glucocorticoids, and progestins in vascular smooth while GPER is only sensitive to androgens. The ability of progestins to downregulate estrogen receptors in the endometrium is well known and opposes both endometrial proliferation and carcinogenesis in response to unopposed estrogen [20, 21]. The relationship between glucocorticoids such as dexamethasone and estrogen receptor signaling is less clear, with most studies showing an ability of DEX to inhibit downstream signaling without direct effects on receptor expression [22, 23]. Previous work in prostate cancer cells similarly showed DHT-induced suppression of GPER that occurs through androgen receptor binding to transcription factors Sp1 and Sp3 and prevent their transcription of GPER [24, 25]. This decrease in receptor mRNA most likely lessens the beneficial effects of estrogen on cardiovascular function.

We found that both low and high dose DHT for 4 weeks doubled intracarotid stiffness assessed via PWV in otherwise healthy, adult female mice. PWV is a strong predictor of cardiovascular mortality in patients independent of other traditional cardiovascular risk factors [26]. Whether the DHT-induced arterial stiffening in this study was tied to the increased body mass induced by DHT cannot be excluded. In fact, arterial stiffness is also induced in mice by a high fat diet and similarly occurs in the absence of an increase in blood pressure [27]. While lifestyle interventions such as avoiding smoking, following a healthy diet, and exercising can reduce CVD risk, there is an increased risk secondary to hyperandrogenism because collagen dysregulation may happen independently of weight gain.

In the current study, DHT treatment in 15-week-old female mice for 4 weeks did not impact blood pressure. In contrast, DHT treatment initiated in female Sprague-Dawley rats at 5 weeks of age resulted in elevated mean arterial pressure by 12 weeks of treatment [28]. The difference in initiation time is most likely not the reason for this discrepancy, as a study by the same group starting DHT post-puberty at 7 weeks of age also showed an increase in blood pressure [29]. Therefore, the discrepancy in blood pressure response between the current study and previous studies in rats could be due to either differences in treatment time or species differences in steroid metabolism [30]. Future studies extending our DHT treatment protocol in mice to 12 weeks will be able to ascertain whether arterial stiffness *precedes* hypertension. We also did not observe evidence of cardiac hypertrophy in our study, although testosterone treatment for 11 weeks in ovariectomized female rats increased both blood pressure as well as cardiac hypertrophy [31]. Therefore, while we find arterial stiffness as an early indicator of cardiovascular damage in mice, treatment for longer periods most likely would increase other risk factors such as hypertension and cardiac remodeling.

The increased arterial stiffness in the current study was not linked to changes in vascular geometry or smooth muscle content but was associated with a decrease in collagen. Dysregulation of collagen is often linked to aging-induced arterial stiffness, but these studies commonly indicate that an *increase* in vascular collagen deposition is the culprit [32, 33]. Both in the current study and in our previous work, collagen does not increase along with PWV and instead many times decreases [14, 15, 34]. This discrepancy in our work versus others may indicate the presence of significant sex differences in the mechanisms of arterial stiffening.

A review of clinical data on the effects of testosterone administration in female-to-male transgender patients does not show a significant increase in overall cardiovascular events [35]. While a recent systematic review highlights mixed results, some individual studies find that androgen therapy in transitioning patients significantly increases arterial stiffness as well as carotid intima-media thickness [36–38]. Similarly, hormone therapy in the female-to-male transgender population increases the incidence of PCOS, resulting in a higher risk for obesity and associated cardiometabolic disturbances [39]. The impact of testosterone on arterial remodeling is most likely impacted by biological sex as well as the presence or absence of other circulating hormones [40, 41].

## Conclusions

This study has important implications for not only PCOS patients but also women taking androgens for fitness, gender transitioning, or reduced libido. The most recent clinical guidelines recommend against prescribing androgen therapy to women for cardiovascular, metabolic, or general well-being and only indicates its use for hypoactive sexual desire disorder [42]. The rationale for these recommendations is mostly due to a lack of well-defined guidelines and symptoms for these other uses along with a lack of data on the efficacy and safety in otherwise healthy women. Along with the findings from the current study, this indicates an important need for further research on the long-term vascular effects of testosterone treatment in women as well as strategies to protect these patients.

## Data Availability

The datasets used during the current study are available from the corresponding author on reasonable request.

## Abbreviations

ddPCR: droplet digital PCR
DXA: dexamethasone
DHT: dihydrotestosterone
ERα: estrogen receptor alpha
GPER: G protein-coupled estrogen receptor
MPA: medroxyprogesterone acetate
PCOS: polycystic ovarian syndrome
